# New Drugs on the Block: Dietary Management and Nutritional Considerations During the Use of Anti-Obesity Medication

**DOI:** 10.3390/nu18060962

**Published:** 2026-03-18

**Authors:** Eleni C. Pardali, Kalliopi K. Gkouskou, Christos Cholevas, Dimitrios Poulimeneas, Kyriaki Tsiroukidou, Dimitrios G. Goulis, Maria G. Grammatikopoulou

**Affiliations:** 1 Immunonutrition Unit, Department of Rheumatology and Clinical Immunology, Faculty of Medicine, School of Health Sciences, University of Thessaly, Biopolis, GR-41223 Larissa, Greece; 2 Department of Biology, Medical School, National and Kapodistrian University of Athens, Goudi Campus, GR-10679 Athens, Greece; gkouskoukal@med.uoa.gr; 3 GENOSOPHY P.C., GR-10444 Athens, Greece; 4 Department of Clinical Pharmacology, Faculty of Medicine, Aristotle University of Thessaloniki, GR-54124 Thessaloniki, Greece; ccholevas@auth.gr; 5 Department of Nutritional Science and Dietetics, School of Health Sciences, University of the Peloponnese, GR-24100 Kalamata, Greece; dpoul@hua.gr; 6 Department of Nutrition and Dietetics, Harokopio University, 70 El. Venizelou Avenue, Kallithea, GR-17671 Athens, Greece; 7 Pediatric Endocrinology Unit, 3rd Department of Pediatrics, Hippokration General Hospital of Thessaloniki, Aristotle University of Thessaloniki, GR-54124 Thessaloniki, Greece; 8 Unit of Reproductive Endocrinology, 1st Department of Obstetrics and Gynecology, Medical School, Aristotle University of Thessaloniki, GR-54124 Thessaloniki, Greece

**Keywords:** GLP-1, GIP, tirzepatide, liraglutide, semaglutide, retatrutide, Ozempic, Wegovy, Mounjaro, Zepbound, amylin, cagrilintide, CagriSema

## Abstract

Incretin-based pharmacotherapy has rapidly transformed obesity management. However, despite its efficacy, gastrointestinal (GI) adverse events (AEs) are common and represent a major driver of treatment discontinuation. Symptoms such as nausea, vomiting, acid reflux, diarrhea, and constipation, not only impair the quality of life, but also compromise adherence, thereby limiting the real-world effectiveness of these agents. Targeted nutritional strategies may play a pivotal role in mitigating these symptoms and supporting sustained treatment. However, most clinical trials have relied on generalized lifestyle advice combined with hypocaloric dietary prescriptions, with limited integration of structured, mechanism-based nutritional counseling tailored to the physiological actions of glucagon-like peptide-1 receptor agonists (GLP-1 RAs) and dual glucose-dependent insulinotropic polypeptide (GIP)/GLP-1 RAs. Consequently, practical guidance for clinicians and dietitians remains fragmented. The present review synthesizes the available evidence on GI AEs associated with incretin-based therapies and examines whether structured, targeted nutritional management can meaningfully reduce symptom burden. We also outline key monitoring strategies and focus on important clinical aspects for physicians and dietitians, aiming to optimize patient outcomes. In addition, we provide detailed information on the spectrum of GI AEs to guide effective management and limit intolerance. By bridging pharmacology with applied clinical nutrition, we aim to provide a pragmatic framework for improving tolerability, sustaining adherence, and translating trial efficacy into durable real-world effectiveness.

## 1. Introduction

The therapeutic landscape of obesity has been reshaped by the rapid integration of incretin-based pharmacotherapies. Glucagon-like peptide-1 analogs and receptor agonists (GLP-1 RAs), such as semaglutide (SEM), dulaglutide (DUL), and liraglutide (LIR), along with the dual glucose-dependent insulinotropic polypeptide (GIP)/GLP-1 receptor agonist tirzepatide (TZP), are widely approved for the treatment of obesity and type 2 diabetes (T2DM) [1,2,3,4,5,6]. Across pivotal trials, the average reported reduction in body weight ranged between 15% and 20% [7,8,9].

More recently, the advent of dual and triple agonists has further intensified expectations, with early data suggesting even greater efficacy, while attempting to maintain acceptable tolerability profiles [8,10]. Combination therapies such as CagriSema (SEM plus the long-acting amylin analogue cagrilintide) have also been developed to simultaneously target GLP-1 and amylin receptors [11,12]. Amylin is a peptide hormone co-secreted with insulin from pancreatic β-cells, in response to nutrient intake. It plays an important role in satiety signaling, gastric emptying, and postprandial glucose regulation [13]. The new drug builds on the hypothesis that complementary physiological actions of amylin and GLP-1 pathways may provide synergistic effects on appetite regulation and body weight reduction, with recent trials demonstrating greater weight loss compared with GLP-1 RA therapy alone [11,12].

Despite these unprecedented benefits, gastrointestinal (GI) adverse events (AEs) remain the principal barrier to long-term adherence to incretin-based therapies [14]. Individuals who develop moderate-to-severe nausea, vomiting, diarrhea, or abdominal discomfort are significantly more likely to discontinue therapy [15]. In particular, patients with T2DM showed a 38% higher likelihood of discontinuing treatment with SEM, LIR, or TZP, whereas among patients without T2DM, the likelihood was 19% [15]. Real-world evidence suggest that as many as two-thirds of patients may interrupt treatment within the first year; notably, re-initiation is more common among individuals who did not experience pronounced GI intolerance during the early phases of therapy [15,16,17]. Furthermore, although dual- and triple-agonist approaches achieve greater body weight reduction, emerging data indicate that this efficacy may be also accompanied by a greater frequency of GI AEs in people with obesity (PwO) and T2DM [18].

Many of these barriers can be mitigated by incorporating a structured, evidence-based lifestyle and nutritional framework alongside incretin pharmacotherapy. However, clinicians frequently lack practical, mechanism-driven guidance on how to translate dietary principles into strategies that directly target delayed gastric emptying, enhance satiety signaling, and reduce oral intake. Integrating pharmacological therapy with individualized nutritional support aims to minimize AEs, preserve nutritional status, and improve persistence, which could ultimately translate into substantial long-term healthcare savings [19]. Thus, the present review aims to synthesize all available evidence and expert perspectives on the best nutritional approaches for the prevention and management of GI AEs and the ideal dietary plan for patients on these new anti-obesity drugs, aiming to support clinicians and dietitians by providing a pragmatic roadmap for safer, more efficient, and sustainable treatment.

## 2. Materials and Methods

For this narrative review, a comprehensive search was conducted using a combination of MeSH terms and free-text keywords at the PubMed and clinicaltrials.gov databases. Citation tracking was also performed using the keywords “GLP-1,” “GIP,” “semaglutide,” “liraglutide,” “tirzepatide,” “retatrutide,” “diet,” and “nutrition.” The search was limited to articles published in the English language with no date restrictions.

The figure was created by the authors using Canva (Canva Pty Ltd., Sydney, Australia) software, with individual graphical elements used in accordance with the Canva Pro licensing terms [20].

## 3. Nutritional Adverse Events

### 3.1. Gastrointestinal Adverse Events and Underlying Causes

GLP-1 RAs exert their metabolic effects by enhancing glucose-dependent insulin secretion, suppressing glucagon, and delaying gastric emptying [21]. The latter contributes substantially to early satiety and is considered the principal driver of GI intolerance [22]. Mechanistically, GLP-1 RAs reduce antral contractions, increase the pyloric tone, and enhance gastric accommodation, thereby slowing the transit of nutrients from the stomach to the small intestine [23,24]. In addition, GLP-1-induced increases in gastric volume are mediated in part through vagal pathways linking the GI tract to central neural circuits, regulating appetite and GI function [25]. Interestingly, gastric emptying has been shown to be approximately 20–30% faster in PwO and in individuals with T2DM compared with healthy individuals [26,27]. Central mechanisms also contribute to these effects, as the area postrema and nucleus tractus solitarius (AP/NTS) of the hindbrain are critical for mediating the appetite-suppressing and emetic responses to GLP-1 receptor activation [28]. These nuclei integrate vagal afferent signals from the GI tract and circulating hormonal signals, thereby linking peripheral GLP-1 activity to central pathways regulating satiety and nausea [29].

The GI tract is the organ system most frequently affected by AEs associated with GLP-1 RA therapy, with reports indicating that up to 80% of treated individuals may experience some degree of intolerance, regardless of the severity [30,31]. Nausea, vomiting, and reduced appetite are particularly common during treatment initiation or dose escalation [5], especially because most individuals may be accustomed to large or frequent meals. Furthermore, the rapid pharmacokinetic profiles of short-acting agents have been proposed to accentuate these effects [32]. Short-acting agents may more readily cross the blood–brain barrier, thereby activating central GLP-1 receptors and exacerbating symptoms such as nausea and anorexia compared to DUL and SEM [33].

Although it was hypothesized that GIP receptor agonists might attenuate GI symptoms, clinical experience with TZP has not demonstrated consistently lower rates than trials using selective GLP-1 RAs [33]. Similarly, trials of the triple agonist retatrutide confirmed that GI AEs remain an important issue, particularly at higher doses and during drug escalation phases [10]. Nevertheless, most events are considered as being of mild-to-moderate severity, tend to diminish over time, and may be mitigated by slower titration or lower starting dose, due to tachyphylaxis [34]. Although still in phase 2 trials, GI AEs were also common in the maridebart cafraglutide (MariTide) trial [35]. AEs, however, were less frequent when a lower starting dose was selected, or when dose escalation was induced.

Similar GI AEs have also been reported in recent CagriSema trials [36]. The occurrence of GI AEs as a whole was greater in the Cagrisema, as compared to the SEM arm [36].

GLP-1-based therapies may also influence intestinal motility and secretory function, contributing to altered bowel habits, including diarrhea [37]. Regarding intestinal obstruction, some studies suggest a possible correlation between GLP-1 RA treatment and its occurrence [38], while others did not report this association, especially among patients with inflammatory bowel disease (IBD) [39,40]. Constipation has been frequently reported, particularly with SEM intake [41], possibly reflecting reduced overall food volume, lower fiber intake, and diminished colonic stimulation.

Emerging evidence from systematic reviews further indicates that GLP-1 RAs increase the risk of cholelithiasis by 50% and induce a 2-fold increase in the risk of gastroesophageal reflux disease (GERD) [42]. Rapid weight loss is a recognized contributor to gallstone formation; thus, the pronounced reductions in body weight induced by these therapies may amplify this risk, particularly among patients with impaired gallbladder motility [43]. Regarding pancreatitis, the available evidence remains controversial, with a meta-analysis failing to demonstrate a definitive association [44].

Hypoglycemia is uncommon among patients on GLP-1-based therapies, particularly when the drugs are used as monotherapy. Hypoglycemia is most frequent among individuals with T2DM who are receiving concomitant insulin or insulin secretagogues such as sulfonylureas [2,3].

### 3.2. Discontinuation Due to Adverse Events

Studies evaluating the dual agonist TZP have revealed a high frequency of GI AEs [32,45], including nausea, dyspepsia, diarrhea, vomiting, abdominal distension and discomfort, eructation, and abdominal pain. These AEs accounted for the majority of treatment discontinuation cases [8,46,47,48,49,50,51,52,53,54] among PwO. Furthermore, the severity of AEs and risk of discontinuation were dose-dependent, with greater TZP doses inducing more severe AEs and a higher possibility of stopping therapy [52,54]. Among participants taking 15 mg of TZP, the discontinuation rate was 10% [48,49,50,51,52,54]. Collectively, these findings indicate that addressing GI AEs may enhance long-term patient compliance and optimize treatment outcomes.

For SEM, withdrawal rates generally range from 1% to 7%, with greater rates being observed at higher doses [7,55,56,57,58,59,60]. On the other hand, LIR at a dose of 3 mg daily has been linked to discontinuation rates ranging between 3% and 10% [9,61,62]. Pharmacovigilance analyses have indicated an elevated reporting frequency of GI disorders across the class, with nausea, vomiting, diarrhea, and constipation being among the most frequently observed symptoms [14].

Differences in tolerability profiles have also been observed among these agents. SEM intake appears to be more frequently associated with nausea, vomiting, constipation, and diarrhea, whereas LIR is associated to a relatively greater reporting rate of abdominal pain [14,41,63]. Regarding the comparison of TZP and SEM, the head-to-head trial data from SURPASS-2 revealed the following GI event percentages: 17–22% vs. 18% for nausea, 13–16% vs. 12% for diarrhea, and 6–10% vs. 8% for vomiting for TZP and SEM, respectively [48].

### 3.3. Nutritional Status and Body Composition

#### 3.3.1. Lean Body Mass

Beyond GI intolerance, appetite suppression and spontaneous caloric restriction induced by these agents raise important concerns regarding possible reductions in lean body mass. Rapid weight loss, insufficient protein intake, and inadequate resistance activity may amplify this effect, underscoring the need for nutritional strategies that actively preserve muscle and skeletal integrity during therapy [64,65,66]. While absolute lean mass typically declines with treatment [7,64], some analyses indicate that the relative proportion of fat-free mass may remain stable [67]. However, maintaining proportionality can obscure clinically relevant losses in functional capacity, particularly in older adults and those at risk of sarcopenia. For example, in the Semaglutide Treatment Effect in People with obesity 1 (STEP-1) trial, participants achieved an average weight loss of 13.6 kg, of which approximately 8.3 kg (62%) involved fat mass, while 5.3 kg (38%) represented lean body mass, including skeletal muscle and other non-fat tissues [7]. Similarly, pooled data from the SURMOUNT-1 trial indicated an absolute reduction in total lean mass of 8.5% across doses [8]. Modeling studies suggest that muscle loss may differ by sex, with women losing approximately 10–15% of their total body weight as lean mass, whereas men appear to be losing 20–25% of their initial body mass, particularly in the absence of structured resistance or strength training programs [68].

Nevertheless, these findings may have important clinical implications for individuals with sarcopenic obesity, a phenotype characterized by the coexistence of excess adiposity and reduced muscle mass or function [69]. In such populations, additional reductions in lean tissue during pharmacologically induced weight loss may exacerbate functional decline, impair mobility, and increase the risk of frailty, particularly among older adults [70,71]. Accordingly, when these therapies are used in populations at higher risk of muscle loss, attention to adequate protein intake and regular resistance-based physical activity may help mitigate potential reductions in muscle mass and support the preservation of functional capacity during weight loss [72].

#### 3.3.2. Osteoporosis

In PwO, intentional weight loss of approximately 7–10% is linked to greater bone turnover and loss of bone mineral density (BMD) [73]. Incretin-based pharmacotherapies may exacerbate this vulnerability through the combination of rapid loss of body mass, reduced mechanical loading, and potential alterations in nutrient intake. Both SEM and TZP have been associated with a higher risk of osteoporosis, or fragility fractures [74,75]. In line with these findings, the regulatory documentation for Wegovy^®^ (SEM) notes a greater incidence of hip and pelvic fractures compared to placebo [2]. Although causality remains difficult to establish and the absolute event numbers are small, these observations reinforce the importance of proactive nutritional and lifestyle strategies aimed at preserving bone density.

## 4. Nutritional Strategies Implemented in Major GLP-1/GIP Trials

In the SURMOUNT-3 and 4 trials, TZP was compared to placebo “as an adjuvant to a reduced-calorie diet and increased physical activity” [46,53] (Table 1). In addition, the SURMOUNT-3 trial included a 12-week lead-in intensive lifestyle intervention, involving a low-calorie diet, physical exercise, and weekly counseling before the beginning of the trial. The recommended energy intake was 1200 kcal/day for the women participants and 1500 kcal/day for the men, and all were allowed to consume up to two liquid meal replacements per day [46]. However, adherence to the diet and/or physical activity prescription was not evaluated. Furthermore, dietary intake of participants was not recorded, although many AEs were associated with GI issues, ultimately leading to treatment discontinuation.

In the large SURMOUNT, STEP, and STEP Teens trials, pharmacotherapy was consistently delivered alongside lifestyle interventions provided by health care professionals, typically including advice on following a healthy dietary pattern with an approximate 500 kcal/day energy deficit and a goal of at least 150 min of physical activity per week [8,30,53,82]. The STEP-3 trial further intensified this model by combining SEM 2.4 mg with comprehensive behavioral therapy (30 counseling sessions) and a structured dietary prescription that relied on meal replacements (1000–1200 kcal/day) during the first 8 weeks, followed by 1200–1800 kcal/day of conventional foods for the remainder of the 68-week intervention [56]. Despite this high level of support, treatment discontinuation, primarily attributed to GI AEs, remained notable. Withdrawal rates were approximately 7% in the STEP-1 trial, where participants received counseling, and 5.9% in STEP-3, despite the addition of intensive behavioral therapy (IBT) and partial meal replacement. The relatively modest difference between these approaches suggests that conventional healthy eating advice alone may be insufficient to fully address medication-related intolerance. A recent trial evaluating TZP, initiated at 2.5 mg and escalated to 10 and 15 mg once weekly, compared two hypocaloric dietary strategies in PwO: low-energy ketogenic therapy (~1200 kcal/day, <30 g carbohydrate, 43% protein, 44% fat) vs. a conventional balanced low-calorie diet (50% carbohydrate, 20% protein, 30% fat) [83]. Despite achieving similar total body weight loss (~10%), the ketogenic approach resulted in greater fat mass reduction and significantly better preservation of fat-free mass, muscle strength, and resting metabolic rate than the carbohydrate-based approach. Appetite suppression was reported more frequently in the ketogenic arm (60% vs. 27%), suggesting a potential interaction between macronutrient composition and incretin-mediated satiety signaling [83].

The Satiety and Clinical Adiposity—Liraglutide Evidence (SCALE) trial, which evaluated LIR, incorporated structured lifestyle counseling, typically prescribing an energy deficit of approximately 500 kcal/day with macronutrient targets of approximately 30% fat, 20% protein, and 50% carbohydrates, together with a recommendation for at least 150 min of physical activity per week [9,62,80]. The SCALE-IBT trial extended this approach through more intensive behavioral treatment, including weight-based caloric prescriptions consistent with the US Department of Agriculture guidance and progressive physical activity goals, increasing to 250 min per week [80]. Despite these comprehensive efforts, GI AEs remained common, occurring in 71.1% of participants receiving LIR compared with 48.6% of those in the placebo group [80]. Additional LIR studies have also provided lifestyle and behavioral advice [64,81]. In addition, a recent trial on retatrutide included a lifestyle intervention; however, AEs increased in a dose-dependent manner [10].

In contrast, the SURPASS trial of TZP did not describe a standardized or drug-specific dietary protocol within the trial publications [47,48,49,50,51,52]. In addition, other randomized controlled trials (RCTs) failed to include dietary information [76,77].

Finally, with regards to the more recent REDEFINE-1 and REDEFINE-2 trials using CagriSema, lifestyle interventions were implemented alongside the pharmacological treatment; however, no detailed information regarding the specific components of these interventions was publicly provided [11,12]. What is known is that the prescribed caloric deficit ranged between 500 and 700 kcal/day.

Overall, most landmark incretin trials incorporated some degree of dietary and physical activity counseling. However, GI AEs consistently persisted and remained the predominant cause of treatment discontinuation. This recurring observation implies that conventional calorie deficit models, even when intensive, may not sufficiently accommodate the unique physiological consequences of GLP-1/GIP RAs. Thus, nutritional management may require a more tailored approach related to the mechanisms of delayed gastric emptying, enhanced satiety signaling, and reduced food intake that characterize these therapies.

### Dealing with Adverse Events

In the SURMOUNT-3 and 4 trials [46,53], GI symptoms were managed through dietary counseling (not-other-defined), symptomatic medications, according to each investigator’s discretion, or by skipping a single treatment dose, as described in the protocol. Similarly, in the STEP and SCALE trials, mitigation strategies primarily relied on gradual dose escalation and supportive lifestyle advice. Although participants received recommendations to follow hypocaloric diets and increase physical activity, structured instructions targeting the physiological mechanisms underlying GI intolerance were not included in the protocols. Consequently, nutritional care functioned largely as an ongoing background therapy rather than as an active tool for AE prevention.

## 5. Boosting Endogenous GLP-1 and GIP Secretion

Preclinical studies provide important clues that dietary composition may interact with incretin pharmacometabolism and pharmacokinetics (Table 2). Research on animals has revealed that chronic high-fat feeding increases GIP and GLP-1 secretion, thus promoting the endogenous supply, without increasing body weight [84]. Notably, dietary fat appears to be a primary driver of increased GIP secretion, whereas augmented GLP-1 responses require excess calories rather than fat exposure alone [84]. Specifically, long-chain polyunsaturated fatty acids (LCPUFAs), such as docosahexaenoic acid (DHA, 22:6, *n*-3), α-linolenic acid (αLA, C18:3, *n*-3), and eicosapentaenoic acid (EPA, 20:5, *n*-3), have been implicated as potential mediators of these effects [85]. These observations imply that habitual dietary fat intake may amplify incretin signaling dynamics and potentially interact with drug tolerability, providing a biological rationale for macronutrient manipulation during incretin-based therapies [84].

*In vitro* and animal studies have suggested that certain metabolite byproducts of bacterial fermentation of dietary fiber can additionally stimulate GLP-1 secretion through the GPCR41 and GPCR43 signaling pathways [92,93,94,95]. Whey protein has been shown to enhance GLP-1 secretion in animal models [96] and clinical studies [97]. Consistent with these mechanistic data, research on humans has shown that several dietary interventions, including high-protein meals, plant-based proteins (buckwheat, fava bean, pea, hemp, and lupin) or the intake of non-digestible and fermentable dietary fibers, stimulate GLP-1 levels, more efficiently than processed meat meals [98,99,100,101,102]. In addition, probiotic yogurt, and to a lesser extent, vitamin D-fortified yogurt, significantly improved circulating GLP-1 levels during caloric restriction in humans, indicating that gut-targeted nutritional strategies may enhance endogenous GLP-1 responses in obesity management [103].

Dietary polyphenols may be another nutritional modulator of incretin physiology. In animal models, anthocyanin-rich blackcurrant extract, curcumin, resveratrol, sweet potato leaf extract, and berberine increased GLP-1 production and glucose-stimulated GLP-1 secretion [104,105,106,107,108,109]. Complementing these findings, clinical evidence demonstrates that a polyphenol-rich curry composed of mixed spices and vegetables increases postprandial total GLP-1 levels dose-dependently [110].

Regarding the exogenous supply, TZP consistently reduces total energy intake while shifting consumption away from highly palatable, lipid-rich options toward standard chow, an effect dependent on intact GLP-1 receptor signaling [87]. Importantly, this suppression appeared to be macronutrient-specific, with lipid intake being reduced to a greater extent than carbohydrate consumption [87]. Complementary mechanistic studies indicate that GIP receptor signaling in leptin-responsive neuronal populations is not essential for weight regulation, whereas pancreatic pathways appear more relevant for glycemic outcomes, reinforcing the concept that appetite and food choice effects are largely GLP-1 driven [86].

In contrast, while animal studies have shown that TZP attenuates metabolic adaptation during weight loss, it does not significantly alter metabolic adaptation in humans, although it does increase fat oxidation and reduce energy intake [90]. Pharmacology modifies appetite signaling, substrate utilization, and food reward; however, nutritional management within trials has rarely been designed to leverage these effects. A more precise alignment between diet composition and meal structure may represent an underused opportunity to improve tolerability and sustain adherence.

Finally, recent animal data on CargiSema indicate that approximately 1/3 of the induced weight loss stems from a direct effect on total energy expenditure as well as energy intake inducing a metabolic adaptation [89].

## 6. Real-World Data on the Dietary Intake of PwO on GLP-1/GIP RA Therapy

Research suggests that individuals using GLP-1 RAs reduce the quantity of food they purchase, with grocery spending falling by approximately 5.3% in the first six months after adoption [111]. The largest declines were concentrated in calorie-dense, highly palatable categories, such as chips and savory snacks, sweet bakery items, and cookies, consistent with reduced appetite and fewer cravings [111,112,113,114]. Complementary evidence suggests that users may also shift the composition of purchases towards more unprocessed foods and modestly higher protein intake, alongside reductions in calories, sugar, saturated fats, and refined carbohydrates [112]. After discontinuation, spending patterns revert to pre-adoption levels, with little evidence of persistent change, implying that much of the observed shift reflects pharmacological effects rather than durable preference transformation [111,115]. Analysis of dietary quality revealed that the healthy eating index (HEI) was poor, regardless of calorie consumption [113]. Individuals on GLP-1 RAs tended to consume less fruit and high-quality protein sources, have an unfavorable fatty acid profile, and have a tendency for caloric and protein intake to be concentrated in the evening [113].

PwO on GLP-1 RAs have been reported to consume adequate amounts of B-vitamins, Copper, Phosphorus, Selenium, and Zinc [116]. However, dietary deficiencies are also apparent, reflecting an inadequate intake of dietary fiber, Calcium, Iron, Magnesium, Potassium, Choline, folate, and vitamins A, C, D, and E [116,117]. Furthermore, reduced appetite and the resulting caloric restriction may further contribute to the development of nutrient deficiencies [114,117]. PwO on incretin-based therapies fail to meet their protein requirements although they appear to consume excessive amounts of fat, including saturated fat [116]. Complementing these observations, nutritional analyses from the SURMOUNT trials also identified reductions in circulating vitamin B12 and D levels [118]. However, these observations were not of sufficient magnitude to result in overt malnutrition or treatment discontinuation in the aforementioned trial. It is important to note that micronutrient status was not systematically or routinely monitored within the trial protocols, limiting firm conclusions regarding the true prevalence and clinical relevance of these deficiencies [118].

GI AEs may further compromise nutrient absorption and exacerbate nutrient deficiencies leading to hair loss, fatigue or low energy, headaches, and changes in skin elasticity [119].

## 7. Dietary Strategies for the Prevention of Gastrointestinal Adverse Events

**Figure 1:**
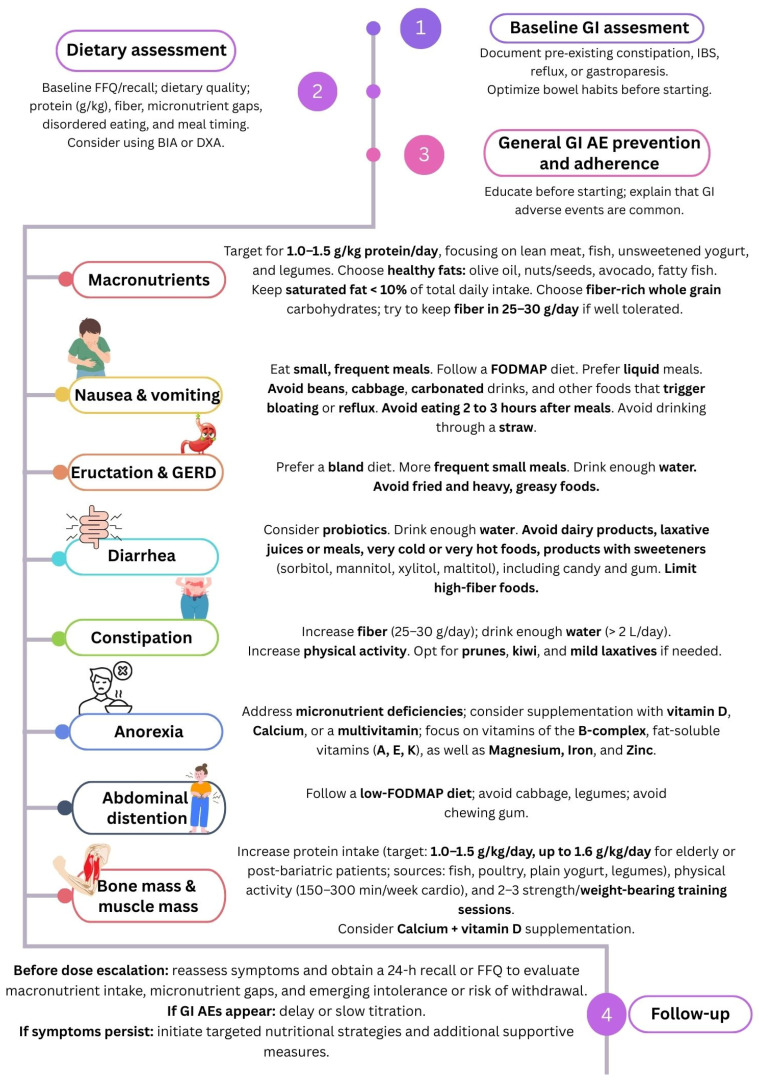
Guidelines for minimizing the severity of GI AEs. AEs: adverse events; FFQ: food frequency questionnaire; FODMAP: fermentable oligosaccharides, disaccharides, monosaccharides, and polyols; GI: gastrointestinal.

Table 3 details the evidence-based interventions that can reduce the severity and frequency of GI AEs. Prior to the initiation of GLP-1 or GLP-1/GIP RA therapy, patients should ideally receive counseling from a registered nutritionist/dietitian (RDN). Education should cover the mechanism of action of the medication, dosing schedules, available formulations, and the central role of nutrition in both optimizing therapeutic outcomes and minimizing intolerance. This early discussion also creates an opportunity to identify pre-existing GI disorders, current symptom burden, or disordered eating patterns that could potentially worsen after treatment initiation [120]. Patients should be reassured that GI symptoms are common, generally mild to moderate, frequently transient, and often improve with simple behavioral adjustments. Self-monitoring of the dietary intake using a diary or electronic diaries (e.g., MyFitnessPal) and reviewing several days of records with a healthcare professional or dietitian can enhance adherence, facilitate early recognition of nutrient inadequacies, and allow timely modification of eating patterns to improve tolerability [121]. Figure 1 illustrates the proposed stepwise approach for the assessment, monitoring, and mitigation of GI AEs during incretin-based therapy. PwO should be followed up before dose adjustment to discuss their symptoms and provide at least one 24-h diet recall or complete food frequency questionnaires (FFQs) to assess macronutrient intake and possible low micronutrient consumption [113].

To reduce GI AEs, it is recommended to limit water intake during meals, by consuming drinks at least one hour before and one hour after each meal [32]. On the other hand, meals should include water-rich foods; however, they must be smaller in volume. In addition, switching from solid to liquid foods may improve tolerability [144]. In contrast, a low-residue diet may also help, particularly during the initiation or dose escalation phases, when gastric accommodation is mostly challenged [32]. Emphasis should be placed on slow eating, the consumption of smaller portions, stopping when reaching satiety, and avoidance of overeating [32,122].

The BRAT (bananas, rice, applesauce, and toast) diet has long been used to treat nausea and vomiting in hospital settings and can be applied to acutely relieve a person from these symptoms. On the other hand, following a bland diet [149], with easily digestible foods that are soft, low in dietary fiber content, without spices, and cooked well, can also prove useful [140] for a longer period of time. Oral nutrient supplementation with vitamin B6 and ginger has also proven useful for treating nausea and vomiting during pregnancy [141], chemotherapy [142,150], and post-surgery [151]. Ginger and peppermint teas may also prove beneficial for some patients [143]. PwO should be advised to avoid drinking through a straw, as this may increase air swallowing and exacerbate GI symptoms [32]. In the event of severe GI AEs, dose escalation should be considered [32].

Symptoms of diarrhea have been shown to improve with the intake of probiotics [134,152]. Similar results have also been reported when the root cause of diarrhea is antibiotic intake [133,153]. Regarding abdominal distention and pain, a low fermentable oligosaccharides, disaccharides, monosaccharides, and polyols (FODMAP) diet may improve similar functional symptoms [127,128]. When small intestinal bacterial overgrowth (SIBO) is suspected, a low-fermentation diet, including low-FODMAP foods is recommended for a period of 4 to 6 weeks, along with meal spacing and avoidance of overnight eating [154]. In addition, probiotics, and sometimes their combination with antibiotics, may also be helpful [148].

Adequate hydration and dietary fiber intake are important strategies for mitigating constipation in patients treated with GLP-1 or GLP-1/GIP RAs. Increasing daily water intake to at least 2 L, combined with the consumption of fiber-rich foods such as kiwi, prunes, or other dried fruits, vegetables, and whole grains, can improve bowel regularity, provided that the fiber is well tolerated [126,129]. Stool softeners may be considered as adjuncts when necessary [122]. In cases of persistent constipation despite these interventions, temporary reduction in the GLP-1 RA dose can be considered [122]. Additionally, maintaining moderate-to-high levels of physical activity may further support GI motility and reduce the risk of constipation [130].

For patients experiencing appetite loss or very low dietary intake, proactive care to ensure micronutrient adequacy is essential. Supplementation with vitamin D, Calcium, and multivitamins should be considered, with particular emphasis on B-complex vitamins, fat-soluble vitamins (A, E, and K), Magnesium, Iron, and Zinc, to prevent deficiencies during periods of reduced caloric intake [124]. Given that obesity can also cause nutrient deficiencies due to altered nutrient metabolism and excretion, oral nutrient supplementation may be important [155] and should be considered on an individual basis.

Dyspepsia and postprandial fullness can be managed by adjusting meal composition and timing. “Lighter”, low-fat, and lower-fiber foods, including soups and well-cooked soft dishes, are better tolerated during flare ups [135]. Alcohol and carbonated beverages should be limited, and patients should consume small, frequent meals and avoid large late-evening dinners. Slow eating, stopping at the point of satiety, and avoiding high-fat, greasy, spicy, or large-volume meals can further reduce gastric discomfort [135].

Specific strategies also target eructation and GI reflux. Limiting foods that promote gas production, including beans, cabbage, and other cruciferous vegetables, and avoiding carbonated drinks are recommended [136]. Adherence to a low-FODMAP diet can be particularly helpful in cases of persistent bloating or gas-related symptoms [136]. For ongoing GERD, small and frequent meals, avoidance of late-night eating, and elimination of trigger foods can significantly reduce the symptom burden [137].

Recent World Health Organization (WHO) guidelines indicate that PwO should receive context-appropriate counseling on behavioral and lifestyle changes as the first step in treatment, including following healthy dietary patterns and adhering to physical activity [156]. In PwO, prescribed GLP-1 or GLP-1/GIP RAs should be provided alongside IBT, entailing goal-setting regarding physical activity levels and dietary intake, restriction of energy intake, counselling (e.g., weekly, 30–45 min), and periodical assessment of goal attainment [156], as follow-ups till 24 months seem to have beneficial effects on body weight, quality of life, and the limitation of AEs. However, these frameworks rarely provide drug-specific instructions on how to eat in the presence of pharmacologically delayed gastric emptying, leaving clinicians without practical tools for the prevention of AEs.

To preserve muscle mass during body weight loss, it is recommended to consume 1.0–1.5 g of high-quality protein per kilogram of body weight, with a higher intake (>1.5 g/kg) considered for older adults, or post-bariatric surgery patients [123]. Protein sources should include lean meats, fish, unsweetened yogurt, and legumes [124], whereas processed meats should be limited [125]. Micronutrient supplementation with vitamin D, Calcium, or a multivitamin is advised, with particular attention to B-complex vitamins, fat-soluble vitamins (A, E, and K), Magnesium, Iron, and Zinc [124].

For individuals at risk of sarcopenia or cachexia, high-protein oral nutritional supplements (15–25 g protein per serving) are recommended, targeting a total daily intake of approximately 1.2–1.6 g/kg of body weight/day from lean sources, ideally combined with resistance training [124,146,147]. To reduce the risk of osteoporosis, Calcium and vitamin D supplementation, along with weight-bearing exercise, is recommended [145,157,158]. A practical goal for patients is 150–300 min/week of moderate-intensity cardiovascular exercise plus 2–3 strength sessions, adjusted for individual fitness levels and tolerability of side effects [159]. Early in therapy, when patients may feel fatigued or nauseated, low-intensity, short-duration walks and light resistance exercise can be initiated and gradually increased as tolerated [32]. Body composition analysis, including bioelectrical impedance analysis (BIA) and dual-energy X-ray absorptiometry (DEXA), is important in evaluating changes in muscle and fat mass.

## 8. Future Research

Personalized medical nutrition therapy (MNT) is increasingly critical for PwO using incretin-based therapies because both body weight loss efficacy and tolerability vary widely, driven in part by genetics, baseline phenotype, and lifestyle. Up to 30–55% of patients on GLP-1 RAs fail to achieve ≥5% weight loss in real-world cohorts [7], highlighting the need for precision lifestyle approaches rather than drug monotherapy alone. Pharmacogenomic studies have shown that variants in *GLP1R* (e.g., rs6923761, rs10305420) and *ARRB1* meaningfully modify glycemic and weight loss responses to GLP-1 RAs, with some genotypes experiencing almost 30% greater HbA1c reduction, while others revealing an attenuated benefit [160]. Emerging pharmacogenomic data and clinical experience support a shift towards tailored genotype- and phenotype-informed MNT alongside GLP-1 RAs to enhance weight loss, improve adherence, and minimize complications [160].

The intestinal microbiota, as a dynamic interface between diet, host metabolism, and pharmacological response, represents a critical, yet underexplored factor in the personalization of obesity treatment [161,162,163]. A deeper understanding of how nutritional strategies interact with GLP-1 and GLP-1/GIP-based therapies to shape the gut microbial ecosystem may provide important opportunities to optimize their efficacy, improve tolerability, and reduce AEs.

New pharmacotherapies targeting incretin pathways are currently underway, including results of the REDEFINE-4 trial, CagriSema and UBT251 injection (NCT07163624). Nevertheless, most ongoing trials have not incorporated structured lifestyle or nutritional interventions within their study protocols [164,165,166].

### Limitations of the Present Review

The present review has some inherent limitations. First, while GI AEs are consistently described as the principal drivers of treatment discontinuation, many RCTs do not provide granular data specifying the exact proportion of withdrawals directly attributable to individual GI symptoms. Moreover, the absence of standardized and validated tools for the routine assessment of gastric emptying and related functional disturbances further complicates their interpretation [167]. This limits the ability to quantify the true burden of intolerance and evaluate which patients might benefit the most from targeted nutritional strategies.

In addition, lifestyle interventions implemented in major trials have usually consisted of general healthy eating advice, or broad caloric prescriptions rather than structured, mechanism-oriented dietary protocols tailored to delayed gastric emptying, early satiety, or reduced intake. Consequently, the real capacity of precision MNT to prevent or attenuate AEs and improve long-term adherence remains uncertain. At the moment, only a limited number of dietary intervention studies report the involvement of a registered dietitian [114]. Well-designed RCTs incorporating standardized, dietitian-led, and symptom-specific nutritional algorithms are needed to determine whether optimized dietary management can meaningfully reduce the discontinuation rates. Another existing gap in the literature concerns body composition outcomes. Many studies have reported total body weight changes without detailed assessments regarding skeletal muscle, functional capacity, or bone parameters. The integration of validated techniques such as BIA and DEXA, alongside strength and performance measures, would allow a clearer determination of the clinical relevance of lean muscle mass and bone changes during therapy.

Finally, the rapid expansion of incretin-based pharmacotherapies requires parallel investment in professional education. Registered dietitians and other healthcare providers must remain up-to-date with the emerging evidence in order to recognize nutritional risks in a timely manner and provide tailored guidance to patients throughout the treatment.

## 9. Conclusions

New pharmacotherapies for obesity have reshaped the therapeutic landscape. Nevertheless, discontinuation rates remain substantial and are predominantly driven by GI intolerance. Addressing these AEs through structured nutritional and dietary management may represent a critical opportunity to improve adherence and long-term effectiveness. Importantly, dietary care should be aligned with the physiological mechanisms of GLP-1/GIP RAs rather than relying exclusively on conventional calorie restriction paradigms. Future investigations should systematically integrate registered dietitians with expertise in incretin-based therapies into clinical pathways and trial designs. Expanding professional education in this area is essential to optimize adherence to treatment and ultimately maximize therapeutic efficacy.

## Figures and Tables

**Table 1:** Characteristics and weight-related outcomes of RCTs evaluating GLP-1 and/or GIP RAs.

Study Name	Author	Design, Blinding	Origin	Duration	Treatment	Patients	BW Related-Outcomes	Lifestyle Management	Discontinuations Due to AEs (Mainly Gastrointestinal)
SURMOUNT-1	Jastreboff [8]	phase 3 RCT, Double-blind	119 sites (9 countries)	72 wks	(i) TZP (5/10/15 mg); (ii) placebo.	N = 2539 adults with BMI ≥ 30 kg/m^2^ or BMI = 27 kg/m^2^ plus a BW-related complication (not T2DM)	Δ% mean BW loss over 72 wk, with dose response	Lifestyle counseling by HCPs to increase adherence to a “healthy” 500 kcal/d deficit diet and ≥150 min of PA/wk	TZP 5 mg: 4.3%; 10 mg: 7.1%; 15 mg: 6.2% (vs. 2.6% placebo)
SURMOUNT-2	Garvey [30]	phase 3 RCT, Double-blind	7 countries	72 wks	(i) TZP (10/15 mg); (ii) placebo.	N = 938 adults with obesity and T2DM	Δ% mean BW loss	Lifestyle counseling by HCPs to increase adherence to a “healthy” 500 kcal/d deficit diet and ≥150 min of PA/wk	4–7% pooled (per general TZP safety)
SURMOUNT-3	Wadden [46]	phase 3 RCT, Double-blind	62 sites (Argentina, Brazil, USA)	2 wk screening; 12 wk lead-in period; 72 wks on treatment	(i) TZP (5/10/15 mg); (ii) placebo.	N = 806 adults with BMI ≥ 30 kg/m^2^ or BMI = 27 kg/m^2^ plus a BW-related complication (not T2DM)	Δ% total mean BW loss after 12 wk lifestyle + 72 wk TZP	12 wk intensive lifestyle intervention (low-calorie diet, PA, counseling) before randomization	TZP 10.5% vs. 2.1% placebo
SURMOUNT-4	Aronne [53]	phase 3 withdrawal RCT, Open-label Double-blind	70 sites (4 countries)	36 wks	(i) TZP (MTD 10/15 mg).	N = 630 adults with BMI ≥ 30 kg/m^2^ or BMI = 27 kg/m^2^ plus a BW-related complication (not T2DM)	mean Δ% in BW from wk 36, % of participants at wk 88 who maintained >80% of the BW loss	Lifestyle counseling by HCPs to increase adherence to a “healthy” 500 kcal/d deficit diet and ≥150 min of PA/wk	Lead-in AE discontinuation 7.0%; post-randomization 1.8% (TZP) vs. 0.9% placebo
				52 wks	(i) TZP (MTD 10/15 mg); (ii) placebo.				
	Frias [76]	phase 2b RCT, Double-blind	47 sites (4 countries)	12 wks	(i) TZP (1/5/10/15 mg); (ii) DUL; (iii) placebo.	N = 318 adults with T2DM for >6 mo, not controlled with diet and PA alone	Δ in mean BW and WC	No dietary plan mentioned	4% overall
	Frias [77]	RCT, Double-blind	13 sites (USA)	12 wks	(i) TZP; (ii) placebo.	N = 111 adults with T2DM for >6 mo, not controlled with diet and PA alone	Δ in mean BW and WC	No dietary plan mentioned	3 patients discontinued (1 on placebo, 1 on 12 mg, 1 on 15 mg)
SURPASS-1	Rosenstock [47]	phase 3 RCT, Double-blind	52 sites (India, Japan, Mexico, USA)	40 wks	(i) TZP (5/10/15 mg); (ii) placebo.	N = 1428 adults with T2DM	Δ in mean BW and WC	No dietary advice mentioned	TZP 5 mg: 3%; 10 mg: 5%; 15 mg: 7% (vs. 3% placebo)
SURPASS-2	Frías [48]	phase 3 RCT, Open-label	128 sites (8 countries)	40 wks	(i) TZP (5/10/15 mg); (ii) SEM 1 mg.	N = 1879 adults with BMI ≥ 25 kg/m^2^ and T2DM	Δ in mean BW	No dietary plan mentioned	TZP 5 mg: 4.1%; 10 mg: 4.6%; 15 mg: 9.3% vs. 4.1% on SEM
SURPASS-3	Ludvik [49]	phase 3 RCT, Open-label	122 sites (13 countries)	52 wks	(i) TZP (5/10/15 mg); (ii) insulin degludec.	N = 1437 adults with BMI ≥ 25 kg/m^2^ and T2DM	Δ in BW	No dietary plan mentioned	TZP 5 mg: 7%; 10 mg: 10%; 15 mg: 11% vs. 1% on insulin degludec
SURPASS-4	Del Prato [50]	phase 3 RCT, Open-label	187 sites (14 countries)	52 wks	(i) TZP (5/10/15 mg); (ii) insulin glargine.	N = 1995 adults with BMI ≥ 25 kg/m^2^ and T2DM	Δ in BW	No dietary plan mentioned	TZP 5 mg: 11%; 10 mg: 9%; 15 mg: 11% vs. 5% on insulin glargine
SURPASS-5	Dahl [51]	phase 3 RCT, Double-blind	45 sites (8 countries)	40 wks	(i) TZP (5/10/15 mg); (ii) insulin glargine.	N = 475 adults with BMI ≥ 23 kg/m^2^ and T2DM	Δ in BW	No dietary plan mentioned	TZP 5 mg: 6%; 10 mg: 8.4%; 15 mg: 10.8% vs. 2.5% on insulin glargine
SURPASS-6	Rosenstock [52]	RCT, Double-blind	135 sites (15 countries)	52 wks	(i) TZP (5/10/15 mg); (ii) insulin lispro.	N = 1428 adults with T2DM on insulin glargine	Δ in BW	No dietary plan mentioned	TZP 5 mg: 6%; 10 mg: 8.5%; 15 mg: 8.5% (vs. 2.4% insulin lispro)
STEP-1	Wilding [7]	RCT, Double-blind	129 sites (16 countries)	68 wks	(i) SEM 2.4 mg; (ii) placebo.	N = 1961 adults with BMI ≥ 30 kg/m^2^ or BMI = 27 kg/m^2^ plus a BW-related complication (not DM)	Δ in mean BW, WC, absolute lean mass (kg)	Counselling each 4 wk to increase adherence to a 500 kcal/d deficit diet and ≥150 min of PA/wk	SEM: 7% vs. 3.1% on placebo
STEP-2	Davies [55]	phase 3b RCT, Double-blind, double-dummy	149 sites (12 countries)	68 wks	(i) SEM (1/2.4 mg); (ii) placebo.	N = 1210 adults with BMI ≥ 27 kg/m^2^ and T2DM	Δ in mean BW and WC	Lifestyle counseling by HCPs every 4 wk to increase adherence to a 500 kcal/d deficit diet and ≥150 min of PA/wk	SEM 1 mg: 5%; 2.8 mg 6.2% vs. 3.5% on placebo
STEP-3	Wadden [56]	phase 3a RCT, Double-blind	41 sites (US)	68 wks	(i) SEM 2.4 mg; (ii) placebo.	N = 611 adults with BMI ≥ 30 kg/m^2^ or BMI = 27 kg/m^2^ plus a BW-related complication (not T2DM)	Δ% in mean BW and mean WC	Meal-replacement LCD for 8 wk followed by hypocaloric diet; prescribed PA titrated from 100 to 200 min/wk; structured IBT with 30 RDN visits	SEM 2.4 mg: 5.9% vs. placebo 2.9%
STEP-4	Rubino [57]	phase 3a withdrawal RCT, Double-blind	73 sites (10 countries)	68 wks	(i) SEM 2.4 mg; (ii) placebo.	N = 803 adults with BMI ≥ 30 kg/m^2^ or BMI = 27 kg/m^2^ plus a BW-related complication (not Τ2DM)	Δ% in mean BW and mean WC	Lifestyle counseling by HCPs each 4 wk to increase adherence to a 500 kcal/d deficit diet and ≥150 min of PA/wk	SEM 2.4 mg: 2.4% vs. placebo 2.2%
STEP-5	Garvey [58]	phase 3 RCT, Double-blind	41 sites (5 countries)	104 wks	(i) SEM 2.4 mg; (ii) placebo.	N = 304 adults with BMI ≥ 30 kg/m^2^ or BMI = 27 kg/m^2^ plus a BW-related complication (not Τ2DM)	Δ% in mean BW and mean WC	Lifestyle counseling by HCPs each 4 wk to increase adherence to a 500 kcal/d deficit diet and ≥150 min of PA/wk	SEM 2.4 mg: 3.9% vs. placebo 0.7%
STEP-6	Kadowaki [59]	phase 3a RCT, Double-blind, double-dummy	28 sites (2 countries)	68 wks	(i) SEM (1.7/2.4 mg); (ii) placebo.	N = 180 adults with BMI ≥ 27.0 kg/m^2^ with ≥2 BW-related comorbidities, or BMI ≥ 35 kg/m^2^ with ≥1 BW-related comorbidity	Δ% in mean BW and mean WC	Lifestyle counseling by HCPs each 4 wk to increase adherence to a 500 kcal/d deficit diet and ≥150 min of PA/wk	SEM 1.7 mg: 3%; 2.4 mg: 3% vs. placebo 1%
STEP-7	Mu [60]	phase 3a RCT, Double-blind	23 sites (4 countries)	44 wks	(i) SEM 2.4 mg; (ii) placebo.	N = 375 adults with BMI ≥ 30 kg/m^2^ or BMI = 27 kg/m^2^ plus a BW-related complication (not T2DM)	Δ% in mean BW and mean WC	Lifestyle counseling by HCPs each 4 wk to promote adherence to a 500 kcal/d deficit diet and ≥150 min of PA/wk	SEM 2.4 mg: 1% vs. placebo 0%
STEP-8	Rubino [61]	phase 3b RCT, Open-label	19 sites (USA)	68 wks	(i) SEM 2.4 mg; (ii) LIR 3.0 mg/d; (iii) placebo.	N = 319 adults with BMI ≥ 30 kg/m^2^ or BMI = 27 kg/m^2^ plus a BW-related complication (not T2DM)	Δ in mean BW and WC	Lifestyle counseling by HCPs each 4 wk to encourage adherence to a 500 kcal/d deficit diet and ≥150 min of PA/wk	SEM 2.4 mg: 0.8% vs. LIR 3 mg/d 6.3% vs. placebo 2.2%
STEP-10	McGowan [78]	phase 3, RCT, Double-blind	30 sites (5 countries)	52 wks	(i) SEM 2.4 mg; (ii) placebo.	N = 207 adults with BMI ≥ 30 kg/m^2^ plus prediabetes	Δ% in mean BW	Lifestyle counseling (HCPs) every 4 wk to encourage adherence to a 500 kcal/d deficit diet and ≥150 min of PA/wk. After wk 5 w, patients received healthy lifestyle counseling per standard care for 28 wk off-treatment	SEM 2.4 mg: 6% vs. placebo 1%
	Anyiam [79]	RCT, Open-label	Centre of Metabolism, Ageing, and Physiology, University of Nottingham, UK	12 wks	(i) SEM 0.25 mg escalated to 1 mg; (ii) VLCD of 800 kcal/d; (iii) SEM plus VLCD.	N = 30 adults with BMI = 27 kg/m^2^ plus T2DM	Δ in BW and body composition	In VLCD groups: received 5 food portions/d of 600 kcal plus 200 kcal of vegetables plus RDN guidance	None
SCALE	Pi-Sunyer [9]	RCT, Double-blind	191 sites (27 countries)	56 wks	(i) LIR 3 mg; (ii) placebo.	N = 2487 adults with a BMI ≥ 30 kg/m^2^ or BMI = 27 kg/m^2^ plus a BW-related complication (not T2DM)	Δ in BW and WC	500 kcal/d deficit diet and increased PA	LIR 3 mg: 9.9%; 3.8% on placebo
SCALE IBT	Wadden [80]	phase 3b RCT, Double-blind	17 sites (USA)	56 wks	(i) LIR 3 mg; (ii) placebo.	N = 282 adults with a BMI ≥ 30 kg/m^2^ (not T2DM)	Δ % in BW	Structured counseling visits; caloric deficit (1200–1800 kcal/d) with 15–20% protein, 20–35% fat, remainder CHO; PA start at 100 min/wk moderate intensity; increase every 4 wk toward 250 min/wk	LIR 3 mg: 8.5%; 4.3% on placebo
SCALE Diabetes	Davies [62]	RCT, Double-blind	126 sites (9 countries)	56 wks	(i) LIR (1.8 or 3 mg); (ii) placebo.	N = 846 adults with BMI ≥ 30 kg/m^2^ and T2DM on MET, thiazolidinedione, or sulfonylurea	Δ in BW and WC	Dietary advice: 30% fat, 20% protein, 50% CHO, with a 500 kcal/d deficit and ≥150 min of PA/wk.	LIR 1.8 mg: 8.6%; 3 mg: 9.2% vs. 3.3% on placebo
	Neeland [64]	RCT, Double-blind	University of Texas Southwestern Medical Center	40 wks	(i) LIR 3 mg; (ii) placebo.	N = 185 adults with BMI ≥ 30 kg/m^2^ or BMI = 27 kg/m^2^ plus a BW-related complication (not T2DM)	Δ in VAT, subcutaneous adipose tissue volume, fat-free tissue, WC	Dietary advice: 30% fat, 20% protein, 50% CHO, with a 500 kcal/d deficit and ≥150 min of PA/wk	0% in LIR; 4.3% in placebo
	Halawi [81]	RCT, Double-blind	Mayo Clinic, Rochester, MN, USA	16 wks	(i) LIR 3 mg; (ii) placebo.	N = 40 adults with BMI ≥ 30 kg/m^2^ or BMI = 27 kg/m^2^ plus a BW-related complication	Δ in BW	Dietetic and behavioral advice	none
	Jastreboff [10]	phase 2 RCT, Double-blind	28 sites (USA)	48 wks	(i) RTT (1/4/8/12 mg with initial doses of 2/4 in >1 mg); (ii) placebo.	N = 338 adults with BMI ≥ 30 kg/m^2^ or BMI = 27 kg/m^2^ plus a BW-related complication (not T2DM)	Δ % in mean BW	Lifestyle intervention by RDN/HCP for a healthy diet and PA	RTT 1 mg: 7%; 4 mg (ID, 2 mg): 6%; 4 mg (ID, 4 mg): 9%; 8 mg (ID, 2 mg): 14%; 8 mg (ID, 4 mg): 6%; 12 mg (ID, 2 mg): 16%; placebo 0%.
	Jastreboff [35]	Phase 2 RCT, Double-blind	Multicenter (78 sites)	52 wks	(i) MariTide: (a) 140 mg; (b) 280 mg; (c) 420 mg each 4 wks no escalation; (d) 420 mg each 8 wks no escalation; (e) 420 mg each 4 wks, 4 wk escalation; (f) 420 mg each 4 wks, 12 wk escalation; (ii) placebo.	N = 592 PwO with/without T2DM	Δ % in BW	No dietary plan mentioned	MariTide 140 mg: 13%; MariTide 280 mg: 12%; MariTide 420 mg each 4 wks no escalation: 16%; MariTide 420 mg each 8 wks no escalation: 27%; MariTide 420 mg each 4 wks, 4 wk escalation: 8%; MariTide 420 mg each 4 wks, 12 wk escalation: 8%; vs. 1% on placebo.
REDEFINE-1	Garvey [12]	phase 3a, RCT, Double-blind	Multicenter (22 countries)	68 wks	(i) CS 2.4/2.4 mg; (ii) SEM 2.4 mg; (iii) cagrilintide 2.4 mg; (iv) placebo.	N = 2108 adults with BMI ≥ 30 kg/m^2^ or BMI = 27 kg/m^2^ plus a BW-related complication (not T2DM)	Δ % in BW, *n* achieving ≥5% weight loss	Lifestyle intervention with a caloric reduction of 500–750 kcal/d	CS 2.4 mg/2.4 mg 3.6%; SEM 2.4 mg: 1.3%; cagrilintide 2.4 mg: 1.3%; placebo: 0.6%.
REDEFINE-2	Davies [11]	phase 3a, RCT, Double-blind	Multicenter (12 countries)	68 wks	(i) CS 2.4/2.4 mg; (ii) placebo.	N = 846 adults with BMI ≥ 27 kg/m^2^ and T2DM (HbA1c 7–10%)	Δ % in BW, *n* achieving ≥5% BW loss	Lifestyle intervention with a caloric reduction of 500–750 kcal/d	CS 2.4 mg/2.4 mg 4.8%; placebo: 0.7%.

AE: adverse events; BMI: body mass index; BW: body weight; CHO: carbohydrates; CS: CagriSema (cagrilintide + semaglutide); DM: diabetes mellitus; DUL: dulaglutide; HbA1c: glycosylated hemoglobin; HCP: health care professional; IBT: intensive behavioral therapy; ID: initial dose; LCD: low-calorie diet; LIR: liraglutide; MariTide: Maridebart cafraglutide; MET: metformin; MTD: maximum tolerable dose; PA: physical activity; RDN: registered dietitians-nutritionists; RCT: randomized controlled trial; RTT: Retatrutide; SCALE: Satiety and Clinical Adiposity—Liraglutide Evidence; SEM: semaglutide; T2DM: type 2 diabetes mellitus; STEP: Semaglutide Treatment Effect in People with obesity; TZP: Tirzepatide; VAT: visceral adipose tissue; USA: United States of America; VLCD: very-low-calorie diet; WC: waist circumference; wk: week.

**Table 2:** Animal studies providing evidence for incretin pharmacokinetics and pharmacometabolism.

		Interventions		
First Author	Animal Models	Pharmaco-logical	Dietary	Results
Akinde-hin [86]	KO mice	individual or dual GIPR and GLP-1R agonists	*ad libitum* intake of a “normal” or HF diet	Single-cell and single-nucleus RNA-seq analyses showed that *Gipr* and *Lepr* are minimally co-expressed in the hypothalamus and hindbrain, whereas substantial co-expression occurs in the embryonic pancreatic endocrine compartment, including α- and β-cells. Accordingly, *Lepr*-specific *Gipr* deletion preserved hypothalamic *Gipr* expression but markedly reduced pancreatic *Gipr* expression. Although GIPR agonism activated cFos in a small subset of POMC neurons, *Lepr-Gipr* KO animals displayed normal BW, body composition, food intake, and energy expenditure under chow and high-fat diet conditions. In contrast, these mice showed improved insulin sensitivity, lower fasting insulin and HbA1c levels, and impaired GIP-stimulated insulin secretion despite unchanged glucose tolerance, indicating a pancreatic contribution to glycemic control. Pharmacologically, acyl-GIP and a GIPR:GLP-1R co-agonist retained BW-lowering efficacy, but the superior glucose-lowering effect of dual agonism was lost in *Lepr-Gipr* KO mice, demonstrating that GIPR signaling in *Lepr*-expressing cells is dispensable for BW regulation but is required for full glycemic benefits in diet-induced obesity.
Geisler [87]	KO mice and rats	individual or dual GIPR and GLP-1R agonists	several choice diet paradigms of chow and a palatable food option	In mice, TZP suppressed TEI while promoting the intake of chow over a high-fat/sucrose diet. GIPR agonism alone did not affect the food choice. The food intake shift observed with TZP was absent in GLP-1R KO mice, suggesting that GIPR signalling does not regulate food preference. TZP also selectively suppressed the intake of palatable food but not chow in a rat two-diet choice model. This suppression was specific to lipids, as GLP-1 RA agonist and dual agonist treatment in rats on a choice paradigm assessing individual palatable macronutrients robustly inhibited the intake of Crisco (lipid) without decreasing the intake of a sucrose (CHO) solution.
Hira [88]	rats	none	standard AIN-93G diet (casein 20–25% wt/wt) or protein-free diet, 10 g/kg	The rats were catheterized in the PV or ILMV. Postprandial glucose levels were higher in the PV group than in the ILMV group, reflecting proximal small intestinal absorption. Active and total GLP-1 levels increased sharply in the ILMV after a protein-containing diet but not after a protein-free diet, suggesting that ileal L cells mediate GLP-1 release. Total GIP levels increased primarily in the PV, consistent with K cell localization in the proximal small intestine. PYY patterns mirrored those of GLP-1. The results validate the use of PV versus ILMV cannulation to distinguish proximal from distal intestinal responses.
Jacobsen [89]	male DIO rats	CagriSema	*Ad libitum* intake of HF-diet	CagriSema induced a 12% BW loss in rats during the experiment Animals treated with the drug ate about 39% less food compared with controls. This indicates that the treatment strongly suppresses appetite. Normally during BW loss, the body reduces EE, making further BW loss harder to achieve. CagriSema blunted this metabolic adaptation, retaining high EE.
Ravussin [90]	mice	TZP	50% calorie restriction	TZP in mice partially prevented the typical reduction in EE that occurs with BW loss, indicating that the drug attenuated metabolic adaptation compared to both vehicle-treated and pair-fed controls. In addition, the respiratory exchange ratio was lower in TZP-treated mice, indicating a shift toward increased fat oxidation relative to CHO use, which likely contributed to the greater BW loss in these animals.
Wang [84]	rats	none	LF diet (4 g of fat/100 g of diet) *ad libitum* for 3 or 13 wk vs. a HF diet (20 g of fat/100 g of diet) in a pair-fed group as control	Chronic HF feeding increased postprandial incretin secretion, independent of obesity. In both the 13-wk and 3-wk studies, HF-fed rats consumed more energy than LF but there was no difference in BW, and only modest differences in body fat, allowing assessment of diet effects without obesity as a confounder. Despite similar BW, HF feeding significantly enhanced the lymphatic GIP and GLP-1 responses to a mixed-meal challenge. Pair-feeding experiments demonstrated that greater GIP secretion was driven primarily by dietary fat content, as HF pair-fed animals showed GIP responses comparable to *ad libitum* HF animals despite a matched energy intake with LF controls. In contrast, elevated GLP-1 secretion required excess caloric intake, as GLP-1 responses in HF-fed animals resembled those of LF control animals. These changes occurred without alterations in fasting glucose, insulin, leptin, DPP-IV activity, and intestinal GIP content, indicating that HF feeding alters incretin secretion through enhanced secretory responsiveness rather than through obesity-associated metabolic disturbances.
Zhang [91]	mice	Acyl-GIP (central i.c.v. and peripheral s.c.), GLP-1/GIP dual agonist, GLP-1 RA	HF diet-induced obesity; chow controls	CNS-*Gipr* KO mice were protected from DIO and glucose intolerance, showing reduced BW and food intake without changes in their energy expenditure. Acute and chronic acyl-GIP administration reduced body weight and food intake and increased cFOS activation in hypothalamic feeding centers. These anorectic and weight-lowering effects were absent or blunted in CNS-*Gipr* KO mice, demonstrating their dependence on central GIPR signaling. Peripheral acyl-GIP partially retains its weight-lowering effects via non-CNS mechanisms (reduced metabolizable energy). Importantly, GLP-1/GIP dual agonism lost its superior efficacy over GLP-1 alone in CNS-*Gipr* KO mice, indicating that central GIPR signaling mediates the enhanced metabolic potency of the dual agonists.

BW: body weight; CHO: carbohydrate; CNS: central nervous system; DIO: diet-induced obesity; DPP-IV: Dipeptidyl peptidase-4; EE: energy expenditure; GIP: glucose-dependent insulinotropic polypeptide; GIPR: glucose-dependent insulinotropic polypeptide receptor; GLP-1: Glucagon-Like Peptide-1; GLP-1 RA: glucagon-like peptide-1 receptor agonist; HbA1c: Glycosylated hemoglobin; HF: high fat; ILMV: ileal mesenteric vein; KO: knockout; LF: low fat; PV: portal vein; POMC: Pro-opiomelanocortin; PYY: peptide YY; RNA: Ribonucleic acid; TEI: total energy intake; TZP: Tirzepatide.

**Table 3:** Possible interventions to reduce GI AEs.

Baseline GI assessment	- Document pre-existing constipation, IBS, reflux, or gastroparesis; optimize bowel habits before starting; avoid GLP-1 in patients with severe gastroparesis. Adopt the three ‘E’s: Education, Escalation, Effective management [122].
Dietary assessment	- FFQ/diet recall; diet quality; protein (g/kg of BW), fiber, micronutrient deficiencies, meal timing [113]. - Screening for binge eating disorder, anorexia nervosa, bulimia nervosa, and night eating disorder [119]. - Track food intake [121]. - Consider assessing of muscle mass (e.g., BIA, DEXA) [119].
General GI AE prevention	- Educate before starting; explain that nausea, vomiting, diarrhea, constipation are common, usually mild/transient; emphasize slow eating, consuming small portions, stopping when full, and avoiding overeating [32,122].
Dose escalation and switching	- Use gradual, individualized titration; pause or step down dose when GI AEs occur; consider switching to more tolerable agent (e.g., DUL, oral SEM) if persistent [32].
Macronutrient intake	- Protein: minimum of 60 g protein/d; target: 1.0–1.5 g/kg of BW; consider >1.5 g/kg of BW for older or post-bariatric surgery patients [123]. Opt for lean meat, fish, unsweetened yogurt, and legumes instead. Limit processed meat [124]. Individualized treatment is required for renal impairment [125]. - Healthy fats: Choose olive oil and plant-based oils, nuts/seeds, avocado, and fatty fish. Saturated fat intake should be <10% of the total daily intake. Limit butter, palm oil, coconut oil [124] - Whole grains: Opt for fiber-rich whole-grain carbohydrates. Limit refined grains (white rice/bread, pasta, and pastries) [124]. - Fiber: 25–30 g of fiber/d [126] from colorful whole fruits and non-starchy vegetables. Sugary juices, canned goods, and sauces should be limited [124]. - Dietary patterns: Mediterranean diet, DASH diet, and plant-based high-protein diet [124].
Micronutrient intake	- Micronutrients: Consider supplementation with vitamin D, Calcium, or a multivitamin; focus on B-complex vitamins, fat-soluble vitamins (A, E, and K), Magnesium, Iron, and Zinc [124].
Abdominal distention and pain	- Low-FODMAP diet [127,128]. - Avoid cruciferous vegetables (e.g., cabbage) and carbonated drinks. - Avoid using a straw for drinks consumption. - Avoid chewing gum.
Anorexia	- Address micronutrient deficiencies; consider supplementation with vitamin D, Calcium, or a multivitamin; focus on vitamins of the B-complex, fat-soluble vitamins (A, E, K), Magnesium, Iron, and Zinc [124].
Constipation	- Increase fiber; opt for 25–30 g of fiber/d [126]. - Drink enough water (>2 Lt/d) [129]. - Increase physical activity [130]. - Consider stool softeners [122].
Cholelithiasis	- Avoid crash/very low-calorie diet; maintain moderate fat; monitor high-risk (rapid BW loss, prior stones); consider UDCA in select cases [131].
Diarrhea	- Probiotics [132,133,134]. - Drink enough water; opt for low-sugar electrolytes; limit alcohol, caffeine, sugary/artificially sweetened drinks [124]. - Avoid dairy products, laxative juices or meals, very cold or very hot foods, products with sweeteners (sorbitol, mannitol, xylitol, maltitol), including candy and gum [32]. - Limit high-fiber foods [32].
Dyspepsia	- Prefer lighter, low-fat, lower-fiber textures during flares (soups, soft foods); limit alcohol and carbonated drinks, which can aggravate fullness and reflux [135]. - Small, frequent meals (4–6/d instead of 2–3 large meals); avoid large late dinners; eat slowly, stop at satiety; avoid high-fat, greasy, spicy, or very large-volume meals that worsen gastric retention [135].
Eructation	- Avoid beans, cruciferous vegetables and carbonated drinks [136]. - Follow a low-FODMAP diet [136].
GERD	- Eat small, frequent meals (4–6/d instead of 2–3 large meals) [137]. - Avoid eating 2–3 h post-meals [137]. - Avoid trigger foods (citrus, tomato, fried/greasy foods, coffee, garlic, onions, peppermint, gas-producing foods like beans, broccoli, raw peppers, raw onions, spicy foods, carbonated drinks, alcohol) [137].
Hydration	- Drink enough water and opt for low-sugar electrolytes. Limit alcohol, caffeine, sugary/artificially sweetened drinks [124].
Hypoglycemia	- Review total carbohydrate intake, pattern and timing; adjust insulin; educate on symptoms and rescue carbohydrates, esp. with reduced intake [119].
Ketosis risk	- Include at least 130 g carbohydrates/d; avoid a ketogenic diet without the supervision by an RDN [138].
Nausea & vomiting	- BRAT diet (when acute effects appear) [139]. - Bland diet (easily digestible foods that are soft-consistency, low-fiber, cooked, gentle to the GI tract and usually non-spicy) [140]. - ONS with vitamin B6 or ginger [141,142]. - Consider trying ginger and peppermint tea [143]. - Avoid fried foods and carbonated beverages [124]. - Drink enough water; opt for low-sugar electrolytes; limit alcohol, caffeine, sugary/artificially sweetened drinks [124]. - Avoid drinking by using a straw [32]. - Choose liquid low-fat meals instead of solid meals high in fat [144]. - In case of persistent vomiting/nausea, consider avoiding drinks during meals (opt for 30–60 min before and/or after meals) [32].
Osteoporosis	- Calcium and vitamin D supplementation [145]. - Weight-bearing exercise [145].
Sarcopenia, cachexia	- Consider implementing high-protein ONS (15–25 g of protein per serving) [124]. - Aim for ~1.2–1.6 g/kg of BW/d of protein using lean foods; pair with resistance training [146,147].
SIBO	- Follow a low-FODMAP diet [24]. - Use probiotics; consider additional antibiotics if necessary [148]. - Opt for soluble fiber (oats, psyllium, apples, carrots) [24].

AEs: adverse events; BIA: bioelectrical impedance analysis; BRAT: bananas, rice, applesauce, and toast; BW: body weight; DASH: Dietary Approaches to Stop Hypertension; DEXA: dual-energy X-ray absorptiometry; DUL: dulaglutide; FFQ: food frequency questionnaire; FODMAP: Fermentable oligosaccharides, disaccharides, monosaccharides and polyols; GERD: Gastroesophageal reflux disease; GI: gastrointestinal; GLP-1: glucagon-like peptide-1; IBS: irritable bowel syndrome; ONS: oral nutrient supplement; RDN: registered nutritionist/dietitian. SEM: semaglutide; SIBO: Small Intestinal Bacterial Overgrowth; UDCA: Ursodeoxycholic acid.

## Data Availability

Not applicable.

## Abbreviations

The following abbreviations are used in this manuscript:

αLA	α-linolenic acid
AEs	Adverse events
AP/NTS	Area postrema and nucleus tractus solitarius
BIA	Bioelectrical impedance analysis
BMD	Bone mineral density
BMI	Body Mass Index
BRAT	Bananas, rice, applesauce, and toast
CS	CagriSema
DHA	Docosahexaenoic acid
DUL	Dulaglutide
DEXA	Dual-energy X-ray absorptiometry
EPA	Eicosapentaenoic acid
FFQ	Food frequency questionnaire
FODMAP	Fermentable oligosaccharides, disaccharides, monosaccharides, and polyols
HEI	Healthy eating index
GERD	Gastroesophageal reflux disease
GI	Gastrointestinal
GIP	Glucose-dependent insulinotropic polypeptide
GLP-1 RAs	Glucagon-like peptide-1 analogs and receptor agonists
IBD	Inflammatory bowel disease
LCPUFAs	Long-chain polyunsaturated fatty acids
LIR	Liraglutide
MNT	Medical nutrition therapy
PwO	People with obesity
RCT	Randomized controlled trials
RDN	Registered dietitian/nutritionist
SEM	Semaglutide
SIBO	Small intestinal bacterial overgrowth
T2DM	Type 2 diabetes mellitus
TZP	Tirzepatide
WHO	World Health Organization
